# Effect of *Padina gymnospora* biowaste inclusion on *in vitro* methane production, feed fermentation, and microbial diversity

**DOI:** 10.3389/fmicb.2024.1431131

**Published:** 2024-07-04

**Authors:** Archit Mohapatra, Shraddha Trivedi, Atul P. Kolte, Chaluvanahalli S. Tejpal, Krishnamoorthy Elavarasan, Shalini Vaswani, Pradeep Kumar Malik, Chandragiri Nagarajarao Ravishankar, Raghavendra Bhatta

**Affiliations:** ^1^Indian Council of Agricultural Research (ICAR)-National Institute of Animal Nutrition and Physiology, Bengaluru, India; ^2^School of Sciences, JAIN (Deemed-to-be-University), Bengaluru, India; ^3^Indian Council of Agricultural Research (ICAR)-Central Institute of Fisheries Technology, Kochi, India; ^4^Uttar Pradesh Pandit Deen Dayal Upadhyaya Pashu Chikitsa Vigyan Vishwavidyalaya Evam Go-Anusandhan Sansthan, Mathura, India; ^5^Indian Council of Agricultural Research (ICAR)-Central Institute of Fisheries Education, Mumbai, India; ^6^Indian Council of Agricultural Research (ICAR), New Delhi, India

**Keywords:** archaea, biowaste, metagenome, methane, *Padina gymnospora*, seaweeds

## Abstract

*In vitro* studies were undertaken aiming to study the methane (CH_4_) mitigation potential of biowaste (BW) of *Padina gymnospora* at the graded inclusion of 0% (C), 2% (A_2_), 5% (A_5_), and 10% (A_10_) of the diet composed of straw and concentrate in 40:60 ratio. The chemical composition analysis revealed that the BW contained higher crude protein (CP), neutral detergent fiber (NDF), acid detergent fiber (ADF), and ether extract (EE) than the PF (fresh seaweed, *P. gymnospora*). The concentration of cinnamic acid, sinapic acid, kaempferol, fisetin p-coumaric acid, ellagic acid, and luteolin in BW was 1.5–6-folds less than the PF. Inclusion of BW decreased (*P* < 0.0001) CH_4_ production by 34%, 38%, and 45% in A_2_, A_5_, and A_10_ treatments, respectively. A decrease (*P* < 0.0001) of 7.5%–8% in dry matter (DM) and organic matter (OM) digestibility was also recorded with the BW supplementation. The BW inclusion also decreased the numbers of total (*P* = 0.007), *Entodinomorphs* (*P* = 0.011), and *Holotrichs* (*P* = 0.004) protozoa. Metagenome data revealed the dominance of Bacteroidetes, Proteobacteria, Firmicutes, Actinobacteria, and Fibrobacter microbial phyla. At the phylum level, Euryarchaeota dominated the archaeal community, whereas *Methanobrevibacter* was most abundant at the genus level. It can be concluded that the inclusion of BW in straw and concentrate based diet by affecting rumen fermentation, protozoal numbers, and compositional shift in the archaeal community significantly decreased CH_4_ production. Utilization of biowaste of *P. gymnospora* as a CH_4_ mitigating agent will ensure its efficient utilization rather than dumping, which shall cause environmental pollution and health hazards.

## Introduction

Methane (CH_4_), with the current atmospheric concentration of 1,890 ppb (Dlugokencky, 2021), is the second most potent greenhouse gas after carbon dioxide (EPA, 2023). With an annual increment of 10–13 ppb, the CH_4_ concentration in the atmosphere is continuously increasing (Nisbet et al., 2019). Altogether, natural and anthropogenic sources are annually emitting 558 Tg (teragram) of CH_4_, and simultaneously 548 Tg is being removed through various sinks. Among the anthropogenic sources, agriculture and livestock production are responsible for the large CH_4_ emissions. Enteric fermentation remains one of the largest sources of CH_4_ emissions in the agricultural sector, annually contributing 87–97 Tg (Chang et al., 2019). India is a major hub for the livestock diversity, possessing 535 million livestock, including 192 million cattle, 110 million buffaloes, 74 million sheep, and 149 million goats (GOI, 2019), which are annually accountable for 9.25 Tg of enteric CH_4_ emissions (Bhatta et al., 2019). In addition to its contribution to global warming, CH_4_ emissions from livestock also represent a major production inefficiency, as each liter of emission carries 55 MJ of energy (World Nuclear Association, 2020) away from the animals.

In above context, novel strategies with holistic efficiency were developed by the researchers to mitigate enteric CH_4_ emissions. In past, antibiotics and ionophores were successfully used for CH_4_ mitigation, but due to raising health concerns, many countries have restricted their uses (Palangi et al., 2022). Alternatively, plant secondary metabolites have been used for the mitigation of enteric CH_4_, and many of these plant metabolites, such as tannins (Malik et al., 2017), saponins (Ku-Vera et al., 2020), flavonoids (Ku-Vera et al., 2020), and essential oils (Belanche et al., 2020; Becker et al., 2023) found effective in reducing enteric CH_4_. These secondary metabolites are not essential for the plant growth but also aid in the defense mechanism against insects and pests (Erb and Kliebenstein, 2020). The distribution and availability of plants possessing secondary metabolites effectively mitigate enteric CH_4_ is region and season specific. Therefore, the search for the alternative phyto sources for CH_4_ mitigation is perpetual. The adoption of the CH_4_ mitigation strategy by the stakeholders is contingent upon the inputs cost and the economic status of livestock farmers. The approaches pertaining to the concentrate feeding is most appropriate. Farmers in developing countries with a large population of low- and non-productive animals cannot afford the high cost of concentrate for the sake of mitigating enteric CH_4_ emissions. Therefore, it is necessary to explore the inexpensive phyto-sources that are locally available in adequate quantity for enteric CH_4_ mitigation.

Seaweeds, adequately available worldwide are rich source of bioactive compounds (Lomartire et al., 2022), and have the potential to reduce enteric CH_4_ emissions (De Bhowmick and Hayes, 2023). Globally, 30 million tons of the seaweeds are annually produced (FAO, 2016). *Padina* is a diverse genus of the brown macroalgae affiliated to the Dictyotaceae family and 58 species have been taxonomically classified (Win et al., 2022). *Padina gymnospora* is one of the prominent species present all along the Indian coast and remains unexplored for their chemical and pharmacological properties. The brown seaweeds possess the enteric CH_4_ mitigation potential (Maia et al., 2016). We performed a preliminary study to explore the CH_4_ mitigation potential of *P. gymnospora*. The utilization of seaweeds in nutraceuticals and hydrocolloids industries (FAO, 2016; Charoensiddhi et al., 2017) produce biowaste. For example, the agar extraction generates 45%–50% of the biowaste (Tůma et al., 2020; Cebrián-Lloret et al., 2022). The disposal of biowaste is a big challenge, which can cause environmental issues and health hazards.

With the above background, *in vitro* studies were carried out to analyze the bioactive compounds and compared the CH_4_ mitigation potential of fresh seaweed (PF) and biowaste (BW) of brown seaweed *P. gymnospora*.

## Materials and methods

### Ethical approval

The *in vitro* studies were carried out at the Energy Metabolism Laboratory of Bioenergetics and Environmental Sciences Division of the ICAR-National Institute of Animal Nutrition and Physiology, Bengaluru, India. The experimentation did not directly involve the animals, except the collection of rumen fluid as a microbial inoculum. Animals were handled as per the standard guidelines and ruminal fluid samples were collected after receiving the approval from the Institute Animal Ethics Committee (NIANP/IAEC/1/2019).

### Collection of seaweed, processing, and composition

**FIGURE 1:**
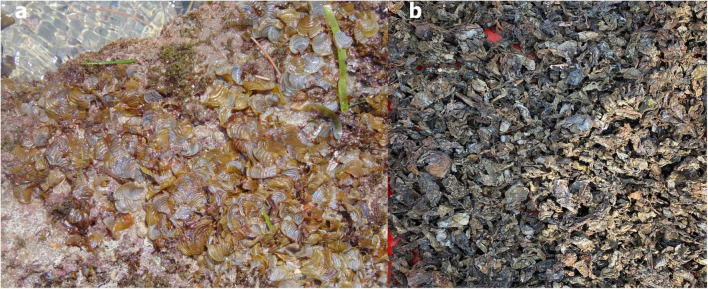
**(a)** Fresh brown seaweed *Padina gymnospora-*PF and **(b)** dried biowaste of *P. gymnospora*-BW obtained after the supercritical fluid extraction.

*Padina gymnospora* was collected from the Indian Ocean along the Mandapam coast in Tamil Nadu, India (Figure 1). The wet biomass of *P. gymnospora* rinsed briefly with the freshwater and sun dried. The dried biomass was then pulverized and selected bioactive compounds such as phloroglucinol, carotenoid, and fucoxanthin were extracted using supercritical fluid extraction technique at ICAR-CIFT, Kochi.

The biochemical composition of PF and BW was analyzed following the standard procedures. The dry matter (DM) content was determined by drying at 80°C for 24 h (AOAC, 2012) and the dried samples were ground using a Cyclotec mill for the further analysis. The total ash content was estimated after incineration in a muffle furnace at 550°C for 4 h and the organic matter (OM) content was computed by subtracting the total ash from the initial dry weight of the sample and expressed as percentage. To determine the crude protein (CP), nitrogen content in the sample was estimated using an automatic nitrogen analyzer (Gerhardt, Germany) and multiplied by 6.25. The fiber fractions such as neutral detergent fiber (NDF) and acid detergent fiber (ADF) were estimated using an automatic fiber analyzer (Fibretherm FT12, Gerhardt, Germany) in accordance with Van Soest et al. (1991)Click or tap here to enter text. The ether extract (EE) was estimated using Soxtherm instrument (Gerhardt, Germany) following the standard procedure of AOAC (2005).

### Bioactive compound analysis

The bioactive compounds in PF and BW were analyzed using LC-MS/MS (liquid chromatography with tandem mass spectrometry, Waters Acuity, USA) at the ICAR-Indian Institute of Horticultural Research, Bengaluru. In brief, sugars were extracted according to Steppuhn and Wäckers (2004) and the extracted samples were filtered and injected into LC-MS/MS following the standard procedure (Arivalagan et al., 2021; Yatung et al., 2023). The phenolic acids and flavonoids were isolated by following the method of Weidner et al. (2000), and Chen et al. (2001), respectively. The drying of ethyl acetate as well as downstream processing was performed (Arivalagan et al., 2021, Yatung et al., 2023) for the phenols estimation and flavonoids as per Bhargav et al. (2018). The organic acids were extracted according to Oliveira et al. (2008) and after processing, the samples were injected into the LC-MS/MS (Yatung et al., 2023). The limit of detection for the phenolic acids, flavonoids, sugars, organic acids, and carotenoids was 0.002, 0.075, 0.01, 0.05, and 0.005 μg/g, respectively. Similarly, the limit of quantitation in LC-MS/MS for the corresponding bioactive compounds was 8.5, 0.25, 0.10, 0.05, and 0.015 μg/g.

### Inclusion of seaweed BW in diet

The diet used in this study consisted of finger millet (*Eleusine coracana*) straw and concentrate mixture in the ratio of 40:60 (w/w). The ratio was decided considering the subsequent animal studies in growing sheep to fulfill their nutritional requirement as per the ICAR feeding standard 2013. A concentrate mixture was formulated using maize grain (320 g/kg), soybean meal (130 g/kg), groundnut cake (120 g/kg), wheat bran (400 g/kg), mineral mixture (20 g/kg), and salt (10 g/kg). Due to the comparable CP (14.6% vs. 13.9%), the wheat bran was partially replaced with BW to formulate the diets for different treatments. The experimental diets (treatments) were prepared by the partial replacement of wheat bran with BW (w/w) to achieve a final inclusion rate of 2% (A_2_), 5% (A_5_), and 10% (A_10_) of the diet, whereas the control (C) treatment was formulated without using BW. Individual ingredients of the concentrate were ground using Cyclotec laboratory mill (CT 293 FOSS) to attain the uniform particle size of 2 mm. Thereafter, the ingredients including BW were mixed in a mixer homogenizer to prepare 100 g of concentrate for each treatment as specified above. Finally, the roughage and treatments specific concentrate were mixed in a homogenizer in 40:60 ratio. The chemical composition of individual feed ingredients and experimental diets was determined.

### Microbial inoculum

Two adult *Holstein Friesian* crossbred bulls (body weight 598.0 ± 15 kg) were used as the donors of ruminal fluid, which served as microbial inoculum. The animals were fed on a diet containing green grass and a concentrate in 70:30. The composition of concentrate mixture was same as defined above under the heading of inclusion of BW in the diet. The rumen digesta, containing both solid and liquid fractions, was collected in the morning at 4 h post-feeding. The collected digesta was stored in a pre-warmed thermos flask and brought to the laboratory while maintaining the temperature (39°C) and anaerobic conditions. The digesta, which had both liquid and solid fractions, was filtered through a single layer of muslin cloth into a preheated thermos flask (39°C) under the continuous flow of carbon dioxide (CO_2_). The rumen fluid was added to a buffered medium maintained at 39°C and stirred continuously with a continuous flow of CO_2_.

### Incubation, total gas, and CH_4_ measurement

For *in vitro* studies, 30 ml of buffered rumen fluid was dispensed with the help of an automatic dispenser (Eppendorf Varispenser, 50 ml) into a 100-ml glass syringe (Haeberle, Germany), which contained a pre-weighed sample (200 mg). For each treatment (C, A_2_, A_5_, and A_10_), three replicates were used in each incubation and total two incubations were performed in succession. The glass syringes were incubated in a *Hohenheim-*type water bath at 39°C for 24 h with periodic automatic shaking. In each incubation, three glass syringes were set as blanks, containing only buffered rumen fluid without feed sample. After 24 h, the incubation was terminated, and the gas volume (ml) from the incubated samples was calculated by difference in initial and final piston position. To cease the fermentation, the syringes were placed on the ice in a large plastic tumbler, and the gas was transferred to the pre-evacuated glass serum vials (10 ml) for CH_4_ analysis.

From the glass serum vials, the individual gas sample was drawn using an airtight glass syringe (Haeberle, Germany). A known volume (0.1 ml) of the gas samples was injected into the gas chromatograph (Agilent 7890B, Germany) equipped with flame ionization detector and Porapak-Q column. The gas chromatography was operated following the conditions described previously (Malik et al., 2017, 2019). The standard of CH_4_ (21.8%, Chemix Specialty Gases and Equipment, Bengaluru) was injected before and after analysis of the actual gas samples. The CH_4_ peak was identified by the retention time and the concentration and volume were calculated with the standard equations as described previously (Malik et al., 2017, 2019).

### CH_4_ production kinetics

For CH_4_ production kinetics, a known quantity of sample (100 mg) was weighed and placed in a glass syringe as described above. For each treatment (C, A_2_, A_5_, and A_10_), three replicates for each time point were used in each incubation and total two incubations were performed in succession. The volume of gas was recorded at 3, 6, 12, 24, 48, and 72 h post-incubation by terminating the fermentation. The gas production (ml) was calculated by difference between the final and initial piston positions, and the gas sample was individually transferred into a 10 ml pre-evacuated glass serum vials. The CH_4_ analysis was performed as described in the previous section. The kinetics of CH_4_ production were determined by the exponential model.


$$G=A\,\left[1-e^{-c\,\left(t-l\right)}\right]$$

Where *G* represents cumulative gas production at time *t*, *A* is the asymptotic gas production, *c* is the rate of gas production per hour, l is the discrete lag time prior to gas production and e is the exponential. The half-time (*t* ½, h) of the degradable fraction was calculated as the time taken for gas accumulation to reach 50% of the asymptotic value.

### *In vitro* DM and OM digestibility

For determining DM and OM digestibility, 500-mg dried sample was weighed and transferred to a 100-ml glass syringe (Haeberle, Germany). Thereafter, 40 ml of buffered rumen fluid was dispensed in the syringe, and the incubations were performed in triplicate in two successions, as described under incubation, total gas and CH_4_ measurement section. Thus, each treatment had six biological replicates, three in each incubation. On the termination of fermentation, the gas was removed from the syringes, and the remaining content was transferred to pre-weighed fiber bags (ST100, Gerhardt) through filtration. The fiber bags containing undigested fractions were repeatedly rinsed with clean water before placing in a hot air oven for drying at 80°C for 24 h. The *in vitro* DM digestibility (IVDMD) was determined using the following formula of Goering and Van Soest (1970).


$$IVDMD\left(\%\right)=\frac{\begin{array}{c} I\,n\,i\,t\,i\,a\,l\,w\,e\,i\,g\,h\,t\,o\,f\,s\,a\,m\,p\,l\,e\,\left(m\,g\right)-\\ W\,e\,i\,g\,h\,t\,o\,f\,r\,e\,s\,i\,d\,u\,e\,\left(m\,g\right) \end{array}}{I\,n\,i\,t\,i\,a\,l\,w\,e\,i\,g\,h\,t\,o\,f\,s\,a\,m\,p\,l\,e\,\left(m\,g\right)}\times 100$$

The total ash in the dried sample was determined by incinerating in a muffle furnace at 550°C for 4 h, and then the OM was calculated by difference as described previously under the chemical composition section. The difference in the OM of initial dried sample and after incubation was considered to be digested, and the *in vitro* organic matter digestibility (IVOMD) was calculated using the following equation:


$$IVOMD\left(\%\right)=\frac{\begin{array}{c} I\,n\,i\,t\,i\,a\,l\,O\,M\,i\,n\,s\,a\,m\,p\,l\,e\,\left(m\,g\right)-\\ O\,M\,i\,n\,r\,e\,s\,i\,d\,u\,e\,\left(m\,g\right) \end{array}}{I\,n\,i\,t\,i\,a\,l\,O\,M\,i\,n\,s\,a\,m\,p\,l\,e\,\left(m\,g\right)}\times 100$$

### Volatile fatty acid and ammonia-nitrogen

Incubation fluid samples from the glass syringes (*N* = 6 per treatment) after withdrawal of the gas samples as stated above under incubation, total gas and CH_4_ measurement section, were transferred into a 50 ml falcon tube placed on the ice. The content was equally divided into two sub-sets. The first set was used for the protozoal enumeration, whereas second set was processed for the volatile fatty acid (VFA) and ammonia-nitrogen (N) estimation. The content was centrifuged at 13,400 rpm for 15 min at 4°C and the supernatant was divided into two subsets for the estimation of VFA and ammonia-N. The pellet obtained from the centrifugation and removal of supernatant was used for the DNA isolation. In half of the supernatant fluid, 25% metaphosphoric acid was added in a ratio of 4:1 (v/v) and stored at −80°C till further analysis. The preserved samples were thawed and VFA were estimated using a gas chromatograph (Agilent 7890B, Germany) as per Filípek and Dvořák (2009) by upholding the GC conditions described previously (Malik et al., 2021; Thirumalaisamy et al., 2022). The VFA concentration was quantified in millimoles (mmol) as per the following equation:


$$V\,F\,A\,\left(m\,m\,o\,l\right)=\frac{\begin{array}{c} P\,e\,a\,k\,a\,r\,e\,a\,o\,f\,s\,a\,m\,p\,l\,e\times C\,o\,n\,c\,e\,n\,t\,r\,a\,t\,i\,o\,n\,o\,f\\ s\,t\,a\,n\,d\,a\,r\,d\times d\,i\,l\,u\,t\,i\,o\,n \end{array}}{P\,e\,a\,k\,a\,r\,e\,a\,o\,f\,s\,t\,a\,n\,d\,a\,r\,d}$$

In half of the rumen fluid, a few drops of saturated HgCl_2_ was added before preserving the samples for ammonia-N. The ammonia-N was determined by following the method of Conway (1957). In brief, 1 ml of boric acid indicator was carefully pipetted into the inner compartment of the Conway dish, while an equal amount of saturated sodium carbonate was added to the outer compartment, and 1 ml of ruminal fluid was pipetted into the outer compartment, opposite to the sodium carbonate. The dish was tightly covered with lid, gently mixed, and left undisturbed at 38°C for 1 h. The boric acid solution was titrated with sulfuric acid (0.01 N), and the ammonia-N concentration was determined with the following formula:


A⁢m⁢m⁢o⁢m⁢i⁢a-N⁢(m⁢g/d⁢L)=V⁢o⁢l⁢u⁢m⁢e⁢(m⁢l)⁢o⁢f⁢ 0.01N⁢H2⁢S⁢O4×14V⁢o⁢l⁢u⁢m⁢e⁢o⁢f⁢s⁢a⁢m⁢p⁢l⁢e

### Protozoal enumeration

The protozoa in the incubation rumen fluid (*N* = 6 per treatment) were enumerated as per the method of Kamra and Agarwal (2003). About 1 ml of the rumen fluid was mixed with an equal proportion of formaldehyde (37%) and left undisturbed overnight. The enumeration and morphological identification of the ruminal protozoa were performed under a phase-contrast microscope (Nikon Eclipse, Japan), and the protozoa, based on their morphology and presence of cilia, were classified under *Entodinomorphs* and *Holotrichs* as per Hungate (1966). The numbers of protozoa in the rumen fluid were enumerated with the following equation:


$$N=\frac{n\times A\times D}{a\times v}$$

Where *N* was the number of protozoa (cells) per ml of rumen fluid, *n* was the average cell count per microscopic field, *A* was the area of the slide on which the diluted rumen fluid sample was spread, *D* was the dilution, *a* was the area of the microscopic field, and *v* was the volume of rumen fluid in the cavity. The protozoal numbers were expressed as ×10^7^ (total protozoa and *Entodinomorphs*) or ×10^6^ (*Holotrich*s) cells/ml.

### Statistical analysis

Data from the study were analyzed in one-way ANOVA using the following model


$$Y_{i-l}=\mu +a_{i}+\varepsilon_{i\,l}$$

Where *Y*_*i*__–__*l*_ is the effect, μ is the mean of the particular level, *a*_*i*_ represents the levels (*i* = 0, 2, 5, and 10 are the supplementation levels of the biowaste and ε_*il*_ is the error. Tukey’s *post-hoc* test was used to examine the significant difference at *P* ≤ 0.05.

### DNA isolation, library preparation, and shotgun sequencing

The pellet was used for the isolation of genomic DNA (*N* = 6 per treatment) using RBB + C method (Yu and Morrison, 2004). The quality of the DNA was confirmed using 0.8% agarose gel electrophoresis, and the concentration was determined by Qubit 4.0 (ThermoFisher Scientific, USA).

The DNA samples were sequenced at Clevergene Biocorp, Bengaluru, India, for the whole metagenome using Illumina HiSeq 2500 platform. The quality of the demultiplexed paired end sequences was assessed using FastQC (v0.11.8; Andrews, 2010) and low-quality bases, adapter contamination, and shorter sequences were removed using Trimmomatic (v.0.39, Bolger et al., 2014) with the following parameters ILLUMINACLIP:TruSeq3-PE-2.fa:2:30:10 SLIDINGWINDOW:15:30 MINLEN:100 TRAILING:30 AVGQUAL:30. The host genomic contamination was removed by mapping and removing the quality curated reads that could map against the reference cattle genome assembly ARS-UCD1.2 (GCF_002263795.1) using Bowtie2 v.2.2.5 (Langmead and Salzberg, 2012). The cleaned reads were uploaded to the Bacterial and Viral Bioinformatics Resource Center (BV-BRC 3.30.19; Olson et al., 2023) server for taxonomic classification. Taxonomic assignments were performed against the Kraken2 Standard database containing the Refseq bacterial, archaeal, viral, plasmid, human, and UniVec core genomes and the resultant Kraken output files (full reports) were parsed through the Pavian (version 0.8.4) online version (Breitwieser and Salzberg, 2020). Pavian was used to construct the composition table of the bacterial and archaeal abundances at the phylum, family, and genus levels. The microbial abundance data was visualized and statistically compared on MicrobiomeAnalyst 2.0 (Lu et al., 2023). The richness and evenness of the microbiome were evaluated using Shannon’s diversity index and beta diversity by Bray–Curtis dissimilarity matrices. The significant difference among the treatments was calculated by non-parametric Kruskal–Wallis rank rum tests. The significant difference between the treatments was ascertained using the Dunn *post-hoc* test (Miles et al., 2022). All statistical analyses were performed in R studio (2023.06.1).

## Results

### Chemical composition

The chemical composition data of feed ingredients and dietary treatments are presented in Tables 1, 2. The compositional data revealed that the CP, fiber fractions NDF and ADF, and EE were considerably higher in BW. However, the total ash in BW was almost half of that in PF. The chemical analysis of dietary treatments (C, A_2_, A_5_, and A_10_) indicated that OM, CP, and EE content were decreased linearly with the increasing levels of PF in the diet. On the contrary, the content of fiber represented by NDF, ADF, and ash were increased with the increasing levels of PF. As compared to the control (C), the OM, CP, and EE were decreased with the graded inclusion of BW.

**TABLE 1:** Chemical composition (g/kg DM) of feed ingredients.

Ingredients	Composition (g/kg DM basis)					
	**OM**	**CP**	**NDF**	**ADF**	**EE**	**TA**
Finger millet straw	900	44.5	655	426	10.9	100
Concentrate	942	178	320	81.2	42.9	58.0
Wheat bran	960	146	386	82.1	42.3	40.0
Seaweed fresh (PF)	528	46	397	363	3.62	472
Seaweed biowaste (BW)	786	139	690	433	11.7	214

g/kg, gram per kilogram; DM, dry matter; OM, organic matter; CP, crude protein (N_2_ × 6.25); EE, ether extract; NDF, neutral detergent fiber; ADF, acid detergent fiber; TA, total ash; PF, *Padina gymnospora* seaweed, fresh without supercritical fluid extraction; BW, *P. gymnospora* seaweed biowaste obtained after supercritical fluid extraction.

**TABLE 2:** Chemical composition (g/kg DM) of dietary treatments.

Composition (DM basis)	Treatments			
	**C**	**A_2_**	**A_5_**	**A_10_**
**Seaweed fresh (PF)**				
OM	923	914	901	873
CP	93.3	86.3	78.4	78.1
NDF	517	534	554	559
ADF	316	316	324	333
EE	22.0	14.6	13.5	10.1
Ash	76.8	85.8	98.5	127
**Seaweed biowaste (BW)**				
OM	923	922	916	901
CP	93.3	91.1	87.7	87.1
NDF	517	536	558	561
ADF	316	316	334	337
EE	22.0	17.7	13.3	13.1
Ash	76.8	78.0	84.0	99.0

C is control without adding the biowaste or fresh seaweed. A_2_ represents the 2% inclusion level of the biowaste (BW) or fresh seaweed (PF) in the diet, whereas A_5_ and A_10_ represent the 5% and 10% inclusion levels of the *P. gymnospora* seaweed biowaste (BW) or *P. gymnospora* seaweed without supercritical fluid extraction (PF) in the diet, respectively. g/kg, gram per kilogram; DM, dry matter; OM, organic matter; CP, crude protein (N_2_ × 6.25); EE, ether extract; NDF, neutral detergent fiber; ADF, acid detergent fiber; TA, total ash; PF, *Padina gymnospora* seaweed, fresh without supercritical fluid extraction; BW, *P. gymnospora* seaweed biowaste obtained after supercritical fluid extraction.

**FIGURE 2:**
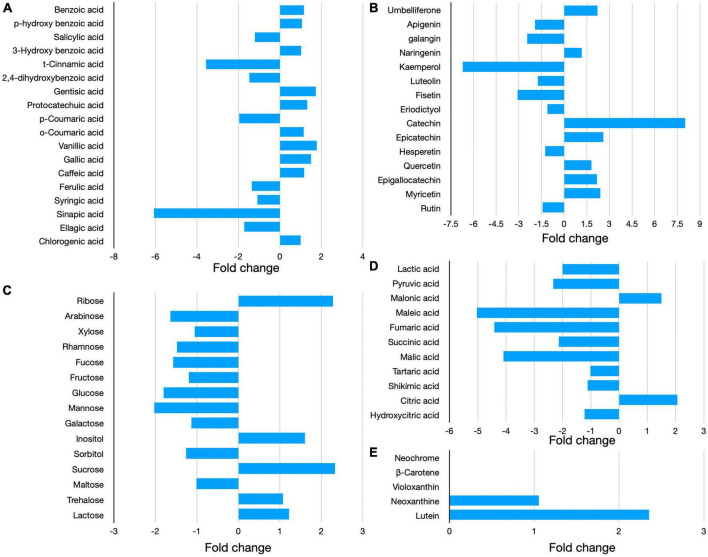
Relative concentration of bioactive compounds, i.e., phenolic acids **(A)**, flavonoids **(B)**, sugars **(C)**, organic acids **(D)**, and carotenoids **(E)** in the biowaste (BW) of *Padina gymnospora*. The folds increase or decrease in the concentration of bioactive compounds in BW is relative to the concentration in PF (seaweed fresh).

Bioactive compounds analysis confirmed the extraction of phenolic acids and flavonoids such as cinnamic acid, sinapic acid, kaempferol, fisetin p-coumaric acid, ellagic acid, and luteolin from the seaweed during supercritical fluid extraction. The data for the individual bioactive compounds in PF and BW is provided in Supplementary File 1. The bioactive compounds having substantial difference in the concentration (1–7.5-folds change) between PF and BW is presented in Figures 2A–E. The concentrations of catechin, epicatechin, epigallocatechin, gallic acid, vanillic acid, and myricetin were higher in BW as compared to PF. The results indicated that the sugars such as mannose, glucose, arabinose, rhamnose, fucose, fructose, and maltose and organic acids such as maleic acid, fumaric acid, and malic acid were extracted to a greater extent from the PF during supercritical fluid extraction. The concentration of ribose, sucrose, inositol, malonic acid, and citric acid were higher in BW as compared to PF.

### CH_4_ production

**FIGURE 3:**
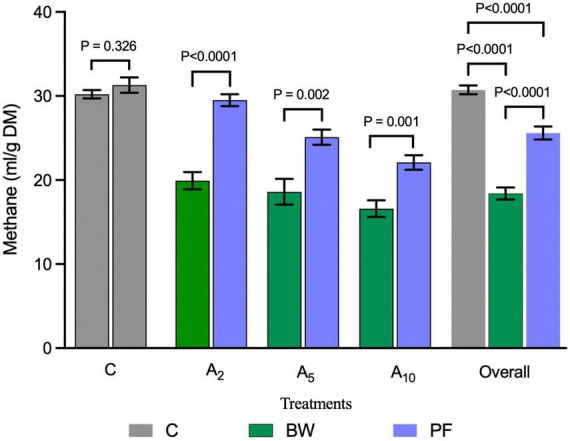
Comparative CH_4_ production from the biowaste of *Padina gymnospora* and fresh seaweed (*P. gymnospora*). C, PF, and BW represent control (without seaweed/biowaste, gray bars), fresh seaweed (blue bars), and seaweed biowaste (green bars), respectively. A_2_, A_5_, and A_10_ represent 2%, 5%, and 10% inclusion of BW or PF in the diet, respectively. Each bar in C, BW, and PF category represented the mean value of six observations (*N* = 6 replicates) for the corresponding treatments C, A_2_, A_5_, and A_10_. Overall categories represent the mean values of average CH_4_ production in A_2_, A_5_, and A_10_ in C, BW, or PF. CH_4_, methane; ml, milliliter; g, gram; DM, dry matter. *P* values were calculated using unpaired parametric *t*-test at 95% confidence level and the significance was ascertained at *P* < 0.05.

The inclusion of BW at 2% (A_2_), 5% (A_5_), and 10% (A_10_) of the diet led to a significant decrease (*P* < 0.05) of 32%, 26%, and 25% in CH_4_ production as compared to PF at the corresponding levels of incorporation (Figure 3). The inclusion of BW in the diet led to 28% more reduction in CH_4_ production (average of A_2_, A_5_, and A_10_) as compared to the CH_4_ production in PF (Figure 3). The BW inclusion in diet produced 34%, 38%, and 45% less CH_4_ as compared to control (C) in A_2_, A_5_, and A_10_ treatments, respectively (Table 3 and Figure 3). There was no difference in CH_4_ production between the treatments A_2_–A_10_.

**TABLE 3:** Effect of seaweed biowaste on total gas, CH_4_ production, and *in vitro* digestibility.

Attributes	Treatments				SEM	*P*
	**C**	**A_2_**	**A_5_**	**A_10_**		
Total gas (ml/g DM)	211^b^	208^b^	190^a^	185^a^	6.46	<0.0001
CH_4_ (ml/g DM)	30.2^b^	19.9^a^	18.6^a^	16.6^a^	3.03	<0.0001
IVDMD (%)	62.7^c^	59.3^b^	58.7^b^	55.1^a^	1.55	<0.0001
IVOMD (%)	61.7^c^	59.3^b^	58.4^b^	53.9^a^	1.63	<0.0001
CH_4_ (ml/g dig. DM)	48.3^b^	33.6^a^	31.6^a^	30.2^a^	4.18	<0.0001
CH_4_ (ml/g dig. OM)	48.2^b^	34.3^a^	33.8^a^	29.4^a^	4.07	<0.0001

C is control without the seaweed biowaste. A_2_ treatment consisted of 2% of the biowaste of *Padina gymnospora* in the diet, whereas A_5_ and A_10_ consisted of 5% and 10% biowaste of *P. gymnospora* seaweed in the diet, respectively. Mean values bearing a, b, c superscripts in a row differ significantly. SEM, standard error of means; *P*, significance level at 5%; CH_4_, methane; IVDMD, *in vitro* dry matter digestibility; IVOMD, *in vitro* organic matter digestibility; ml/g DM, milliliter per gram of dry matter; ml/g dig. DM, milliliter per gram of digestible dry matter; ml/g dig. OM, milliliter of digestible organic matter.

In this study, BW and PF irrespective of the inclusion levels led to an average reduction of 40% in BW and 16% in PF as compared to treatment C (Table 3). Therefore, the BW at all the inclusion levels, i.e., A_2_, A_5_, and A_10_ was relatively more effective than PF in decreasing CH_4_ production.

### Gas production and digestibility

The effect of BW inclusion at 2% (A_2_), 5% (A_5_), and 10% (A_10_) of the diet on total gas and digestibility are presented in Table 3. Results from the study indicated a decrease (*P* < 0.0001) in total gas (ml/g DM) production due to the graded incorporation of BW at 5% (A_5_) and 10% (A_10_). There was no difference in the gas production between C and A_2_ treatments. Data on digestibility indicated a reduction of 7.5%–8% in IVDMD and IVOMD due to the graded inclusion of BW. The study demonstrated that the IVDMD and IVOMD were negatively correlated (*r*^2^ = −0.88, −0.89) with the inclusion levels of BW (Supplementary File 1) in the diet. There was no difference in the IVDMD and IVOMD between A_2_ and A_5_ treatments. Overall, a negative correlation was observed between the inclusion levels of BW levels and total gas (*r*^2^ = −0.75) or CH_4_ production (*r*^2^ = −0. 07) (Supplementary File 1). There was a positive correlation between CH_4_ and IVDMD (*r* = 0.66) or IVOMD (*r* = 0.63).

Total volatile fatty acid production (mmol) was adversely affected by the BW inclusion at 10% (A_10_) of the diet. A similar decrease in acetate and propionate production was observed in A_10_ treatment (Table 4). There was no difference in the pH due to inclusion of BW in A_2_ and A_5_ treatments, whereas, a significant difference (*P* = 0.002) in pH was observed between C and A_10_ and A_2_ and A_10_ treatments. The BW inclusion did not affect (*P* = 0.042) the ammonia-N concentration. The inclusion of BW in A_5_ and A_10_ treatments decreased the numbers of total (*P* = 0.007), *Entodinomorphs* (*P* = 0.011), and *Holotrichs* (*P* = 0.004) protozoa as compared to treatment C. There was no difference in the numbers of total, *Entodinomorphs*, and *Holotrichs* protozoa between the C and A_2_ treatments (Table 4).

**TABLE 4:** Effect of seaweed biowaste on fermentation characteristics and rumen protozoa.

Attributes	Treatments				SEM	*P*
	**C**	**A_2_**	**A_5_**	**A_10_**		
pH	6.86^a^	6.85^a^	6.90^ab^	6.98^b^	0.029	0.002
Ammonia-N (mg/dl)	13.7	13.4	14.3	14.8	0.312	0.184
TVFA (mmol)	72.2^b^	69.6^ab^	67.6^ab^	63.8^a^	1.77	0.010
Acetate (mmol)	49.4^b^	47.9^ab^	46.8^ab^	43.9^a^	1.16	0.041
Propionate (mmol)	12.6^b^	12.0^ab^	11.5^a^	11.5^a^	0.261	0.020
Butyrate (mmol)	7.18^b^	6.94^b^	6.58^b^	5.79^a^	0.303	<0.0001
Iso-butyrate (mmol)	0.804^b^	0.762^b^	0.753^b^	0.695^a^	0.006	0.0002
Valerate (mmol)	1.29^b^	1.22^b^	1.20^b^	1.09^a^	0.041	<0.0001
Iso-valerate (mmol)	0.850	0.819	0.791	0.789	0.014	0.066
Total protozoa (×10^7^ cells/ml)	19.6^c^	19.1^bc^	14.6^ab^	13.8^a^	1.49	0.007
*Entodinomorphs* (×10^7^ cells/ml)	19.4^b^	18.5^ab^	14.5^a^	13.8^a^	1.40	0.011
*Holotrichs* (×10^6^ cells/ml)	1.63^b^	1.22^ab^	0.510^a^	0.510^a^	0.277	0.004

C is control without the seaweed biowaste. A_2_ treatment consisted of 2% of the biowaste of *Padina gymnospora* in the diet, whereas A_5_ and A_10_ consisted of 5% and 10% biowaste of *P. gymnospora* seaweed in the diet, respectively. Mean values bearing a, b, c superscripts in a row differ significantly. SEM, standard error of means; *P*, significance level at 5%; CH_4_, methane; TVFA, total volatile fatty acids; mg/dl, milligram per deka liter; mmol, millimole.

### Kinetics of CH_4_ production

**FIGURE 4:**
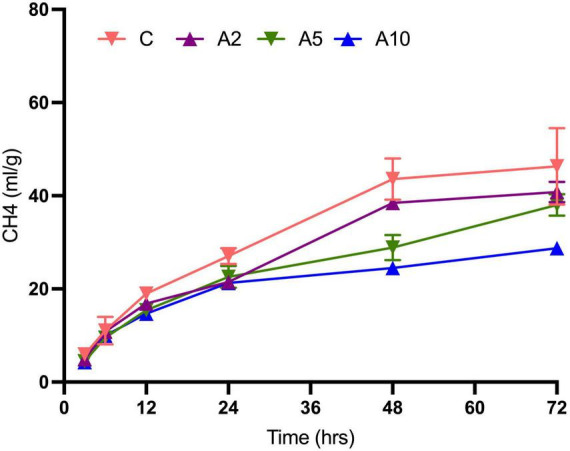
Effect of seaweed biowaste (BW) inclusion levels on CH_4_ production kinetics. C, A_2_, A_5_, and A_10_ represented the treatments with variable levels of BW inclusion of 0%, 2%, 5%, and 10% in the diet, respectively. CH_4_, methane; C, control; ml, milliliter; g, gram; DM, dry matter; hrs, hours of incubation. Each data point in line plot represents the mean value of the six observations (*N* = 6 replicates per treatment) at different incubation hours.

Kinetics study revealed a significant effect of the variable levels of BW and incubation hours on CH_4_ production (Figure 4 and Table 5). In the initial hours of incubation, the CH_4_ production was similar among the treatments. There was a significant difference in CH_4_ production beyond 12 h of incubation in A_5_ and A_10_ treatments. The CH_4_ production within the treatment was affected by the advance incubation time. Data from the study indicated a significant effect of levels x time interaction on CH_4_ production. A significant difference in the CH_4_ production at time zero (“a”) and plateau (“b”) at the infinite times as well as rate constant (c), was observed due to the incorporation of BW at the graded levels (Table 6). The prediction equations developed for predicting CH_4_ production (Y) are given in Table 6.

**TABLE 5:** Kinetics of CH_4_ production as influenced by graded inclusion of seaweed biowaste.

Time (hours)	Treatments				SEM	*P*
	**C**	**A_2_**	**A_5_**	**A_10_**		
3	5.91^v^	4.91^v^	4.46^v^	4.25^u^	0.369	0.208
6	11.03^v^	10.82^w^	9.51^vw^	9.94^v^	0.359	0.652
12	18.98^aw^	16.87^abx^	15.42^bwx^	14.67^bw^	0.948	0.004
24	27.03^ax^	21.52^bx^	22.60^bxy^	21.29^bx^	1.33	0.038
48	43.60^ay^	38.47^ay^	28.88^by^	24.49^by^	4.36	<0.0001
72	46.32^ay^	40.78^ay^	38.03^abz^	28.74^bz^	3.67	0.0005
SEM	6.54	5.96	5.10	3.77	Interaction *P* < 0.0001	
*P*	<0.0001	<0.0001	<0.0001	<0.0001		

C is control without seaweed biowaste, whereas A_2_ consisted of 2% of the biowaste of *Padina gymnospora* in the diet, A_5_ and A_10_ consisted of 5% and 10% biowaste, respectively. SEM is standard error of means and *P* is the significance at 5%. Mean values bearing the superscripts u, v, w, x, y, z compares the means and represent the significance within the column, whereas the superscript a, b compare the mean values and represent the significance within the row.

**TABLE 6:** Prediction equations for CH_4_ production for the control and diets consisted variable levels of seaweed biowaste.

Time (hours)	Treatments			
	**C**	**A_2_**	**A_5_**	**A_10_**
a	1.59	2.51	2.85	0.792
b	51.9	47.9	41.6	27.8
c	0.032	0.027	0.028	0.059
*r* ^2^	0.917	0.923	0.938	0.844
RSD	5.36	4.51	3.46	4.08
Equation (Y)	0.5878 × X + 9.317	0.5150 × X + 8.064	0.4448 × X + 7.589	0.3125 × X + 8.637

C is control without seaweed biowaste, whereas A_2_ consisted of 2% of the biowaste of *Padina gymnospora* in the diet, A_5_ and A_10_ consisted of 5% and 10% biowaste, respectively. RSD is residual standard deviation. Y is CH_4_ production (ml/g) at X time hours. a is CH_4_ production when the x is zero, whereas b and c represent the CH_4_ production at infinite times and rate constant of CH_4_ production, respectively.

### Alpha and beta diversity

**FIGURE 5:**
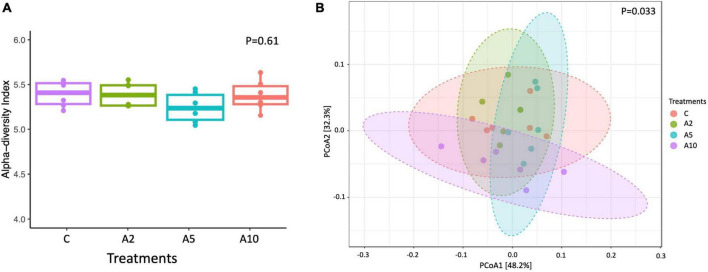
Alpha diversity in different treatments (C, A_2_, A_5_, and A_10_) represented by Shannon index **(A)**, beta diversity index among the treatments **(B)**. C was control (without seaweed biowaste), whereas A_2_, A_5_, and A_10_ treatments represented the inclusion of BW at the corresponding levels of 2%, 5%, and 10% in the diet. Individual mean value in each bar was based on six observations (*N* = 6 replicates).

The bacterial diversity in terms of species richness and evenness represented by Shannon index was similar among the groups (*P* = 0.61; Figure 5). There was a significant difference in archaeal diversity between the groups (*P* = 0.027; Supplementary File 1). The beta diversity determined by Bray–Curtis revealed that the bacterial and archaeal communities among the treatments were different (*P* = 0.03; Figure 5). A significant difference in the microbial communities was observed between A_2_ vs. A_10_ (*P* = 0.035) and A_5_ vs. A_10_ (*P* = 0.032) (Supplementary File 1).

### Metagenome composition

A total 130 GB with average 5.44 GB (range 17.01–19.24 million) paired end sequencing reads per sample were generated. The data quality filtration (Q > 30) and Illumina adapter cleaning resulted in an average removal of 4.18% of sequencing data. Further, ∼0.021% of the sequence data was filtered due to host (*Bos taurus*) DNA contamination.

**FIGURE 6:**
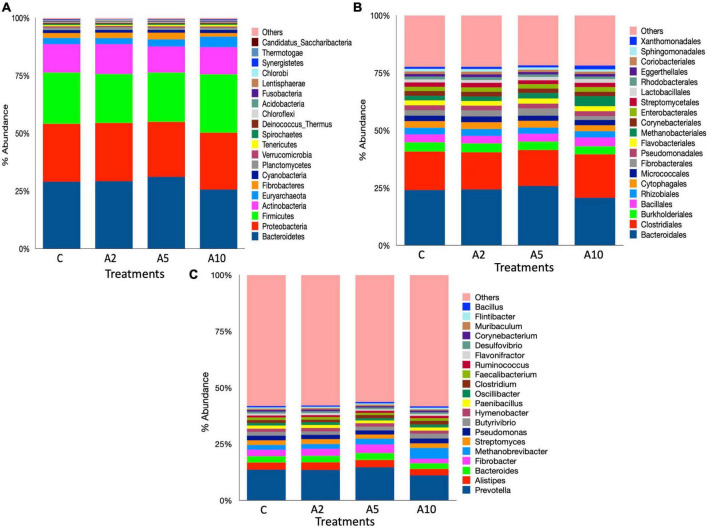
Rumen microbiota at different taxonomic ranks. The stacked bar graphs illustrate the relative percent abundances of rumen microbiota in different treatment as influenced by the biowaste inclusion levels at the **(A)** phylum, **(B)** order, and **(C)** genus levels. The stacked bar graphs represent the top 20 microbes at each taxonomic ranks. C was control (without seaweed biowaste), whereas A_2_, A_5_, and A_10_ treatments represent the inclusion of BW at the corresponding levels of 2%, 5%, and 10% in the diet. Each mean value was based on six observations (*N* = 6 replicates) for the corresponding treatments C, A_2_, A_5_, and A_10_.

A total of 41 phyla, 169 orders, and 1,379 microbial genera were identified in the metagenome (Supplementary File 1). Bacteroidetes, Proteobacteria, Firmicutes, Actinobacteria, Euryarchaeota, and Fibrobacteres were most abundant microbial phyla, aggregately constituted ∼93% of the microbiota (Figure 6). The abundance of Firmicutes was significantly higher (*P* = 0.007) in A_10_ treatment as compared to A_2_ and A_5_, whereas the abundance of Fibrobacteres was significantly lower (*P* = 0.013) in A_10_ treatment compared to A_5_. Similarly, the abundances of Kiritimatiellaeota (*P* = 0.018), Verrucomicrobia (*P* = 0.024), and Lentisphaerae (*P* = 0.033) were also decreased in A_10_ treatment. The abundances of Bacteroidetes, Proteobacteria, Euryarchaeota, and Actinobacteria was comparable among the treatments. The Firmicutes to Bacteroidetes ratio (F/B) was higher in the A_10_ group as compared to other treatments (Supplementary File 1).

**FIGURE 7:**
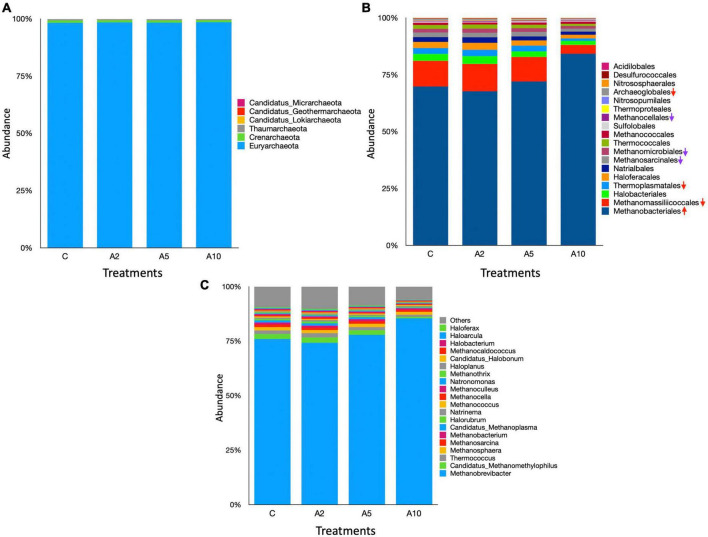
Rumen archaea at the different taxonomic ranks. The stacked bar graphs illustrate the relative abundances of archaea in different treatment as influenced by the biowaste inclusion levels at the **(A)** phylum, **(B)** order, and **(C)** genus levels. The stacked bar graphs represent the top 20 microbes at genus level. C was control (without seaweed biowaste), whereas A_2_, A_5_, and A_10_ treatments represent the inclusion of BW at the corresponding levels of 2%, 5%, and 10% in the diet. Each mean value is based on six observations (*N* = 6 replicates) for the corresponding treatments C, A_2_, A_5_, and A_10_.

The archaeal community comprised 6 phyla, 18 orders, and 109 genera in the metagenome (Supplementary File 1). At the phylum level, Euryarchaeota was most prominent archaea and constituted >98% of the community (Figure 7). The abundance of Euryarchaeota phylum was comparable among the treatments (*P* = 0.857). The archaeal phyla, such as Crenarchaeota, Thaumarchaeota, Candidatus Lokiarchaeota, Candidatus Geothermarchaeota, and Candidatus Micrarchaeota, represented ∼2% of the archaeal community. Among these, the abundance of Candidatus geothermarchaeota was significantly different (*P* = 0.05) between A_2_ and A_10_ treatments.

At the order level, Bacteroidales and Clostridiales dominated the microbiota, represented 21%–25% and 15.5%–19% of the metagenome, respectively. There was no difference in the abundance of Bacteroidales among the treatments (*P* = 0.117). Overall, 28 microbial orders were significantly different among the treatments (Supplementary File 1). The inclusion of BW in A_10_ treatment led to the higher abundance (*P* = 0.0242) of Clostridiales. There was a significant difference in the distribution of Burkholderiales (*P* = 0.009) between C and A_10_ and the distribution of Bacillales (*P* = 0.01) between A_2_ and A_10_ treatments. Methanobacteriales were most abundant archaea at the order lever, constituted ∼70% of the total. A total four archaeal order namely Methanobacteriales (*P* = 0.02), Methanomassiliicoccales (*P* = 0.004), Thermoplasmatales (*P* = 0.04), and Archaeoglobales (*P* = 0.02) were influenced by the BW supplementation.

At the genus level, *Prevotella* was most abundant. The abundance of *Prevotella* was not affected (*P* = 0.107) by the inclusion of BW in this study. The abundances of the other dominated genera *Alistipes* and *Bacteroides* were also comparable among the treatments (Figure 6). The BW inclusion at 10% (A_10_) of the diet led to a significant decrease (*P* = 0.014) in the abundance of *Fibrobacter*, whereas the abundance of *Butyrivibrio* was increased (*P* = 0.014). At the genus level, *Methanobrevibacter* dominated the archaeal community and constituted 75%–85% of the total archaea (Supplementary File 1). The BW inclusion in A_10_ treatment led to a significant increase (*P* = 0.02) in the abundance of *Methanobrevibacter*. The *Candidatus Methanomethylophilus* (*P* = 0.004), *Thermococcus* (*P* = 0.04), *Methanobacterium* (*P* = 0.03), and *Candidatus Methanoplasma* (*P* = 0.004) genera constituted ∼6% of the archaeal community and their abundances were significantly decreased with the inclusion of BW at 10% level (A_10_) of the diet. The abundance of *Methanomassiliicoccu*s, was also adversely affected (*P* = 0.002) by BW supplementation in treatment A_10_.

## Discussion

The removal of a major fraction of sugars and organic acids during the supercritical fluid extraction from PF was primarily responsible for the higher concentration of CP, NDF, ADF, and EE in BW. The washing of waste from the supercritical fluid extraction prior to its use as an anti-methanogenic agent was accountable for the difference in ash and OM content between BW and PF. Seaweeds in general contains considerably higher ash content than terrestrial plants (Rhein-Knudsen et al., 2017). The ash content in the PF is in good agreement with the previous studies (Marinho-Soriano et al., 2006; Pereira, 2016; Rhein-Knudsen et al., 2017). The higher ash content in *P. gymnospora* could be attributed to high absorption and uptake of minerals by brown seaweeds as compared to green and red algae (Siahaan et al., 2018). We have not quantified the divalent cation concentrations in the *Padina*, but it is a well-established fact that the brown seaweeds possesses high concentrations of divalent cations (Manivannan et al., 2008) and SO_4_ (Sasaki et al., 1999). The CP content in *P. gymnospora* was in consonance with the previous reports (Mebeau and Fleurence, 1993; Makkar et al., 2016; Shanmuganathan and Pandima Devi, 2016; Ahmed et al., 2022). In this study, the EE content (3.62%) in PF was in good agreement with the previous studies (Winarni et al., 2021; Kustantinah et al., 2022). The higher EE in BW than the PF could be due to the removal of sugars and organic acids during the extraction of bioactive compounds. The fiber fractions NDF and ADF in this study falls in line with the reported range (Lahaye, 1991; Ahmed et al., 2022). Seaweeds due to the high degree of alginates, differs in composition from that of terrestrial plants and contains high polysaccharide content, and the cell wall fractions (Rogers and Perkins, 1968; Ahmed et al., 2022).

Our study implied that the inclusion of BW at 2% (A_2_), 5% (A_5_), and 10% (A_10_) of the diet led to 32%, 26%, and 25% less CH_4_ production as compared to the inclusion of PF at the corresponding levels in the diet. Our research demonstrated that inclusion of BW at 2% (A_2_), 5% (A_5_), and 10% (A_10_) of the diet was better than the PF in decreasing CH_4_ production at the corresponding levels. Irrespective of the levels, the inclusion of BW in present study decreased CH_4_ production by 28% and 40% as compared to PF and C, respectively. The potential to decrease CH_4_ emissions by using seaweed is currently a much-discussed topic (Vijn et al., 2020; Roque et al., 2021). Seaweed is gaining popularity as a viable animal feed due to its polysaccharides, which have the ability to reduce CH_4_ emissions (Abbott et al., 2020; De Bhowmick and Hayes, 2023). Multiple groups have reported a significant decrease in CH_4_ production due to the use of seaweeds, but the impact of seaweed biowaste obtained after the selective removal of bioactive compounds of nutraceutical interest on CH_4_ production remains unexplored. A very high reduction in CH_4_ production (67%–95%) was reported on using *Asparagopsis*, a red seaweed at a inclusion level of 1%–5% in the diet (Chagas et al., 2019; Roque et al., 2019). Similar to our study, De Bhowmick and Hayes (2023) reported that the inclusion of brown and green seaweeds led to a reduction of 20%–45% in CH_4_ production. In another study, a reduction of 17% in CH_4_ (ml/g dig. OM) was reported on the incorporation of brown seaweed at 5.0% in the diet (Künzel et al., 2022). The reduction in CH_4_ production with the inclusion of BW was consistent with the previous reports, where brown seaweeds were used at the almost similar levels. However, the extent of reduction was less than that reported for the *Asparagopsis*, which could be attributed to the very high content of bromoform in the *Asparagopsis*.

A decrease in CH_4_ production could be an output of the depression in feed digestibility (McSweeney et al., 2001; Tavendale et al., 2005; Malik et al., 2017), shift in the fermentation pattern, change in VFA production, decreasing numbers of protozoa (Malik et al., 2017, 2023; Baruah et al., 2019; Poornachandra et al., 2019), structural and functional changes in the archaeal community (Henderson et al., 2015; Danielsson et al., 2017; Tapio et al., 2017; Martínez-Álvaro et al., 2020). The comparable ammonia-N among the treatments revealed that the inclusion of BW up to 10% in the diet do not interfere with the protein degradation and subsequent microbial protein synthesis. These findings are in good agreement with the findings of Belanche et al. (2016), who did not report any adverse impact of brown seaweed on ammonia-N.

The graded incorporation of BW in the diet decreased IVDMD (6%–12%) and IVOMD (4%–12%). The reduction in digestibility was dose-dependent, and the dry (*r*^2^ = −0.88) and organic matter (*r*^2^ = −0.89) digestibility were inversely correlated with the inclusion level of BW. The reduction in digestibility with the graded inclusion of BW can be attributed to high NDF and ADF content in test treatments (A_2_–A_10_). Moreover, the high content of caffeic acid could also be accountable for the depression in digestibility. In an *in vitro* study, it was demonstrated that the caffeic acid, a typical phenol linearly decreased the DM digestibility when added up to 40 g/kg DM (Jin et al., 2021). The decreased abundance of *Fibrobacter*, a major bacteria accountable for the fiber degradation (Shinkai et al., 2009; Raut et al., 2019) with BW inclusion at the highest level (10%) could also be responsible for decreased IVDMD and IVOMD. Despite the higher abundance of *Butyrivibrio* in BW incorporated treatments, the IVDMD and IVOMD was low, which indicated that the *Butyrivibrio* has a minor role in the degradation of fiber (Teather and Ohmiya, 1991) and metabolizes xylans and pectins (Palevich et al., 2020). Since the xylose sugar was extracted from the PF during supercritical fluid extraction, the inadequate substrate might have hindered the improvement in digestibility by *Butyrivibrio.*

Our results demonstrated that the depression in digestibility was certainly one of the factors accountable for significantly lower CH_4_ production proven by a negative correlation between CH_4_ production and inclusion levels of BW in the diet (*r*^2^ = −0.67). Contrary to our findings, Choi et al. (2020) reported an improvement in DM digestibility on the graded inclusion of brown seaweed *Sargassum fusiforme* at 1%–10% of total ration. This deviation in findings can be attributed to the difference in the concentration of water soluble carbohydrates, which were removed from the PF during supercritical fluid extraction. The compositional difference between *P. gymnospora* and *S. fusiforme* in terms of CP (4.6% vs. 9.07%), NDF (39.7% vs. 19.5%) and ADF (36.3% vs. 13.0%) may also be accountable for the deviation. VFA data also revealed an adverse effect of BW on the rumen fermentation at 2%–10% levels in the diet.

A decrease in the ruminal protozoa held accountable for the mitigation of CH_4_ production in an indirect way by interfering (Poornachandra et al., 2019) with the H_2_ supply to methanogenic archaea (Bhatta et al., 2009; Malik et al., 2017). The interdependency of protozoa and methanogens is a classic example of symbiosis and syntrophy, as the former is ecto- or endo-symbiotically adhered, whereas the latter one received H_2_ supply from the protozoa. Inclusion of BW at different levels in the diet appears to have an inhibitory effect on the protozoa as evidenced by a linear decrease in total, *Entodinomorphs*, and *Holotrichs* protozoa. These findings are in good agreement with Choi et al. (2020), who reported a decrease in protozoa numbers due to the supplementation of brown seaweed, *S. fusiforme*. Flavonoids are polyphenolic bioactive compound known to inhibit protozoa and lowering CH_4_ (Oskoueian et al., 2013). The reduction in ruminal protozoa may also be attributed to the high concentration of some flavonoids, particularly catechin, myricetin, and naringenin in BW. These findings are in consonance with the earlier studies where a decline in protozoal numbers was reported due to catechin (Ramaiyulis et al., 2019), myricetin, and naringenin (Oskoueian et al., 2013). The previous reports suggest that the gallic acid did not affect CH_4_ production (Aboagye et al., 2019; Wei et al., 2019). This could be due to the difference in the nature of tannins as gallic acid belongs to hydrolysable tannins (Patra and Saxena, 2011), which is less effective than condensed tannins in reducing CH_4_ production (Bhatta et al., 2009). The role of other phenolic acids, such as gentisic acid and protocatechuic acid, in CH_4_ reduction is yet to be elucidated. Moreover, the high content of caffeic acid in BW could also be accountable for a linear decrease in CH_4_ production as demonstrated in a study (Jin et al., 2021).

This study fully explored the microbial diversity, as evidenced by the large number of phyla (41), orders (169), and genera (1,379) identified. Metagenome data revealed that Bacteroidetes, Proteobacteria, and Firmicutes were three largest microbial phyla, representing approximately 2/3 of the total ruminal microbiota. Bacteroidetes and Proteobacteria abundances remained unaffected by the graded incorporation of BW, while Firmicutes abundance increased at the highest level of BW incorporation in A_10_ treatment. The metagenome data demonstrated that the bacterial community was far rich than the archaea and this is in good agreement with the report of Henderson et al. (2015). The dominance of Bacteroidetes, Proteobacteria, and Firmicutes in the metagenome is in good agreement with the previous reports (Kim et al., 2014; Söllinger et al., 2018; Malik et al., 2022a,b, 2023).

The results of this study corroborate previous findings that the Euryarchaeota phylum dominated the archaeal community (Yanagita et al., 2000; Kim et al., 2011; Malik et al., 2022b,^2023^). The identification of additional archaeal phyla in the rumen was consistent with the Shin et al. (2004), Xue et al. (2019), and Malik et al. (2022b,2023). Methanobacteriales have been identified as prominent methanogens at the order level, which is in consonance with the previous studies (Seedorf et al., 2015; Malik et al., 2022b; Reichenbach et al., 2024). The anti-microbial and anti-methanogenic effects of flavonoids are known (El-Lakany et al., 1997; Oskoueian et al., 2013). Flavonoids such as myricetin, quercetin, catechin, and naringenin in high concentrations in BW as such (Patra and Saxena, 2011) or new metabolites produced from the metabolic transformation (Simons et al., 2005), affected the *Candidatus Methanomethylophilus*, *Thermococcus*, *Methanobacterium*, and *Methanomassiliicoccus* methanogens. Metagenome data revealed that *Methanobrevibacter* remain unaffected by the flavonoids in BW. The impact of phenolic acid such as gentisic, protocatechuic and chlorogenic acid on the rumen methanogens is not fully understood and need further investigation. Methanogens belonging to Methanomassiliicoccales, Thermoplasmatales, and Archaeoglobales orders exhibited a substantial decrease in the abundances, indicated that the BW inclusion at the highest level in the diet had a detrimental impact on these methanogens. An increase in *Methanobrevibacter* abundance revealed that the BW inclusion did not affect the entire archaeal community in a uniform manner; thus, the niche vacated by the decreased abundances of the above methanogens filled with the *Methanobrevibacter*. The contribution of the above methanogens in CH_4_ production is yet to be elucidated and confirm whether the cumulative production from these methanogens is equal to CH_4_ production by *Methanobrevibacter*.

In this study we have not considered the animal traits, but the increasing abundance of Firmicutes and F/B ratio with BW inclusion might have implications on the feed efficiency (Turnbaugh et al., 2006), average daily gain (Min et al., 2019), and milk fat yield (Jami et al., 2014). The decreased abundances of Methanomasillicoccales and Thermoplasmatales with lesser rumen protozoa and decreased dry matter digestibility cumulatively contributed to the significant reduction in CH_4_ production.

A patent has been applied to the patent office of Government of India claiming the anti-methanogenic properties and levels of feeding of biowaste of *P. gymnospora* (CBR number 39591 dated 9 November 2021).

## Conclusion

It may be concluded that the biowaste obtained after the supercritical fluid extraction of *P. gymnospora* has higher content of protein, fiber and ether extracts and low content of sugar, organic acids, and phenolic compounds than the fresh *P. gymnospora*. The concentration of flavonoids (myricetin, quercetin, catechin, and naringenin), phenolic acids (gentisic, protocatechuic, and chlorogenic), and organic acids (malonic and citric acids) was higher in the biowaste. Our study established that the inclusion of biowaste of *P. gymnospora*, in straw- and concentrate-based diet was better than the fresh seaweed in decreasing CH_4_ production. The graded inclusion of biowaste led to a linear and significant decrease in CH_4_ production with a concurrent decrease in dry and organic matter digestibility, total protozoa, *Entodinomorphs*, and *Holotrich*s. The inclusion of biowaste also led to a compositional changes in the archaeal community. Therefore, the inclusion of biowaste at a level of 2%–10% in the diet can lead to a significant reduction in CH_4_ production in both indirect and direct ways. The utilization of biowaste as CH_4_ mitigating agent in the diet will ensure its efficient use rather than dumping in open which can cause environmental pollution and health hazards. Further studies in ruminants are warranted to confirm the efficacy of biowaste of *P. gymnospora* in CH_4_ reduction in live animal system and validation of the inclusion level at which feed fermentation and nutrient utilization remains unaffected. The specific phenolic acid, flavonoids and other bioactive compounds need to be identified to elucidate the mechanism by which CH_4_ reduction is achieved.

## Data availability statement

The datasets presented in this study can be found in online at www.ncbi.nlm.nih.gov/bioproject/PRJNA1080562 and the other data can be found in the article/Supplementary material.

## Ethics statement

The animal studies were approved by Institute Animal Ethics Committee (NIANP/IAEC/1/2019). The studies were conducted in accordance with the local legislation and institutional requirements. Written informed consent was obtained from the owners for the participation of their animals in this study.

## Author contributions

AM: Investigation, Methodology, Software, Visualization, Writing – original draft. ST: Investigation, Methodology, Visualization, Writing – original draft. AK: Formal analysis, Investigation, Methodology, Software, Visualization, Writing – original draft. CT: Formal analysis, Resources, Writing – review & editing. KE: Formal analysis, Resources, Writing – original draft. SV: Formal analysis, Methodology, Writing – original draft. PM: Conceptualization, Funding acquisition, Project administration, Resources, Supervision, Writing – review & editing. CR: Resources, Supervision, Writing – review & editing. RB: Funding acquisition, Project administration, Resources, Supervision, Writing – review & editing.
